# Two Species of Wild Long-Fruited Jute (*Corchorus olitorius*) Characterization and Phylogenetic Analysis of the Complete Chloroplast Genomes

**DOI:** 10.3390/ijms27125527

**Published:** 2026-06-18

**Authors:** Xingcai An, Guanghui Du, Junyuan Dong, Xia An

**Affiliations:** 1 School of Agriculture, Yunnan University, Kunming 650500, China; xcan2001str@163.com (X.A.); du0425@163.com (G.D.); 13964552682@163.com (J.D.); 2 Zhejiang Xiaoshan Institute of Cotton & Bast Fiber Crops, Zhejiang Institute of Landscape Plants and Flowers, Zhejiang Academy of Agricultural Sciences, Hangzhou 311251, China

**Keywords:** jute, chloroplast genome, wild species, germplasm, genetic diversity

## Abstract

Jute (*Corchorus* spp.) is the most important bast fiber crop, providing raw materials for textiles, bio-composites, and papermaking. This study analyzed the chloroplast genomes of two wild long-fruited jute species: Qiaojianyehuangma (QJYHM) and Maliyehuangma (MLYHM). The chloroplast genomes exhibited typical circular quadripartite structures (LSC, SSC, IRa/IRb), containing 129 genes (37 tRNA, 8 rRNA, 84 mRNA). Overall GC content was 36.76%, indicating high genetic conservation. Compared with cultivated varieties, wild varieties exhibit differences in LSC region length, IR boundary positions, and repetitive sequences, reflecting minor sequence variations in the chloroplast genome that occurred during domestication. Codon preference analysis showed both wild species favor A/U-ending synonymous codons, with a strong preference for methionine’s AUG codon. Repetitive sequence analysis revealed 280 and 252 dispersed repeats in Qiaojianyehuangma and Maliyehuangma, respectively, primarily mononucleotide SSRs. Based on Ka/Ks analysis, it was discovered that most chloroplast genes were under purifying selection. In contrast, positive selection signals were detected in *rpl23*, *ycf1*, and *ycf2*, implying their involvement in adaptive evolution. We identified 161 polymorphic sites (97 SNPs, 64 InDels), with *ycf1* as a mutation hotspot. Phylogenetic analysis clustered both wild species with *Corchorus capsularis* with a 100% bootstrap value, forming a well-supported sister group. This study provides basic chloroplast genome data for two wild *Corchorus olitorius* accessions, revealing their conserved genomic features and minor sequence variations.

## 1. Introduction

Long-fruited jute (*Corchorus olitorius*), an annual herb of genus *Corchorus* in the Malvaceae family, is a vital fiber crop with substantial economic and ecological significance [1,2]. It is extensively cultivated in tropical and subtropical regions [3]. However, for a long time, the breeding of long-fruit jute variants has relied on limited genetic resources, resulting in a steady narrowing of its genetic foundation, and the progression of key agronomic features like stress tolerance and quality is facing bottlenecks [4]. Long-term domestication has led to a narrowing of the genetic base of cultivated jute and a significant decline in stress tolerance, whereas the wild germplasm retains a rich array of stress-tolerance alleles [5]. Compared with cultivated varieties, wild species have significant tolerance to abiotic stresses such as drought and salinity, and are key resources to overcome the bottleneck of stress-resistant breeding of cultivated varieties and broaden their genetic background [6,7].

Regarding the origin and classification of *Corchorus olitorius*, the traditional view is that Africa is its original origin, having later spread to Asia and other regions through human migration and trade [8]. Molecular systematics suggests that there could be two distinct diversification hubs in Africa and Asia [9,10]. *Corchorus olitorius*, native to Northeast Africa, and *Corchorus capsularis*, originating from the South Asian subcontinent, are the two most economically significant cultivated species of the genus *Corchorus* [4,11]. The genetic material of eukaryotic plant cells consists of the nuclear genome and the cytoplasmic genome (plasmid genes), with the cytoplasmic genome further comprising the chloroplast genome and the mitochondrial genome [12]. These three components work together to regulate plant growth and development, material metabolism, and environmental adaptation [13]. The nuclear genome carries the vast majority of genetic information and follows the laws of biparental inheritance [14]; the chloroplast genome has a highly conserved structure, is maternally inherited, and evolves at a stable rate, serving as a core molecular marker for studies on species phylogeny and evolutionary origins. Additionally, constraints imposed by the chloroplast genome are considered a factor in the domestication bottlenecks observed in crops [15,16]. Although the mitochondrial genome is also maternally inherited, it exhibits greater structural variation and an evolutionary rate intermediate between that of the nuclear and chloroplast genomes. It plays an irreplaceable role in elucidating the mechanisms of cytoplasmic male sterility in crops, studying adaptive evolution under stress conditions, and investigating nuclear–cytoplasmic interactions [17].

The chloroplast genome is genetically stable, making it an ideal molecular marker for studies of phylogeny, genetic differentiation, and the origins of domestication. [18,19]. In the family Malvaceae, comparative chloroplast genomic analyses have been instrumental in deciphering phylogenetic relationships among genera and understanding the evolutionary dynamics of species [20,21]. A comparative assessment of chloroplast genomes from cultivated varieties and their wild counterparts can elucidate disparities in genomic architecture, sequence polymorphism, and evolutionary pathways [22,23]. It also provides a scientific basis for analyzing the genetic mechanism in the process of crop domestication and mining key functional genes [24]. Promoting the protection, development, and utilization of wild resource populations is crucial for improving the efficiency of variety improvement and biodiversity research.

While the full chloroplast genome sequence of the cultivated *Corchorus olitorius* has been reported [25], there have been no investigations into the complete chloroplast genome sequence of wild *Corchorus olitorius*. This study aims to elucidate the characteristics of the chloroplast genomes of wild ramie species. Furthermore, this study seeks to provide partial molecular evidence at the organelle level in support of the dual-origin hypothesis of ramie, based on genomic structure, mutation sites, and selective pressures. Potential candidate genes for ramie breeding were identified, which will aid in the effective utilization of wild genetic resources to optimize cultivated varieties.

## 2. Results

### 2.1. Characteristics of the Chloroplast Genome Structure

**Figure 1:**
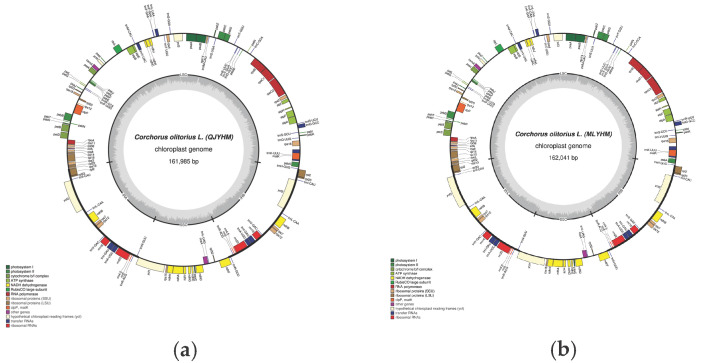
Circular map of QJYHM (**a**) and MLYHM’s chloroplast genomes (**b**). Genes encoded on the reverse strand are located within the interior of the circular map, whereas those encoded on the forward strand are positioned on the exterior. The guanine–cytosine (GC) content is depicted by the dark gray inner circle, while the adenine–thymine (AT) content is illustrated by the light gray circle.

The complete chloroplast genome of *Corchorus* is characterized by a highly conserved circular tetrad structure comprising LSC, SSC, and IR regions, along with consistent functional gene classification (Figure 1). However, interspecific variations are observed in genome size and fine-scale structural features. The chloroplast genome size of two wild jute species measured in this experiment was 161,985 bp (QJYHM) and 162,041 bp (MLYHM), respectively.

Further examination of functional region sizes within the genome revealed slight length variations among the LSC, SSC, and IR regions. The length of LSC, SSC, and IR of QJYHM was 89,520 bp, 20,413 bp and 26,026 bp, respectively, and the length of the MLYHM corresponding region was 89,681 bp, 20,442 bp and 25,959 bp, which showed that the length of LSC and SSC increased and the length of IR shortened (Table 1). This variation pattern aligns with the typical dynamics of IR region expansion and contraction observed during chloroplast genome evolution. Base composition analysis revealed that the two germplasms shared an identical chloroplast genome GC content of 36.76%, reflecting strong homology in their nucleotide composition. The analysis of GC distribution across regions showed that the GC levels in different functional regions showed the same distribution rule, that is, the GC content in the IR region was significantly exceeded those of the LSC and SSC regions, which was closely related to the enrichment of ribosomal RNA genes possessing high GC content in the IR region. At the same time, there was little difference in GC levels across the functional regions within the varieties, and the difference in GC levels in the LSC, SSC, and IR regions was only 0.03%, 0.01%, and 0.08%, respectively, which further supports the chloroplast genome conservation between the two wild *Corchorus olitorius*.

### 2.2. Gene Structure Annotation

The chloroplast genomes of both wild species had 129 genes, including 37 tRNAs, 8 rRNAs, and 84 mRNA genes, with no pseudogenes found. The constancy in gene count across the two wild *Corchorus olitorius* species indicates that their chloroplast genomes are highly conserved. Furthermore, the absence of pseudogenes implies that no functional gene degradation has occurred in the wild *Corchorus olitorius* chloroplast genome, suggesting well-preserved gene integrity.

Further statistical investigation of the chloroplast genes function of two *wild Corchorus olitorius*, as shown in Table 2. The chloroplast genome of wild *Corchorus olitorius* predominantly comprises genes associated with photosynthesis, self-replication, various other functions, and genes of undetermined function. These genes form the genetic foundation for the photosynthetic electron transport pathway and energy metabolism. Genes related to self-replication include ribosomal large and small subunit proteins, RNA polymerase subunits, and abundant rRNA and tRNA genes, which contribute to gene expression regulation and metabolic pathways. Other functional genes in the genome include mature enzymes, proteases, and cytochrome c synthesis genes, all of which play key roles in gene expression control and metabolism. Furthermore, the genome contains conserved putative open reading frames with unknown functions, particularly the *ycf* genes, which are potential candidates for future functional research.

### 2.3. RSCU Analysis

The codon preference RSCU (Relative Synonymous Codon Usage) values of two wild *Corchorus olitorius* were analyzed (Table 3). Analysis revealed that there was little difference in the number of codons and RSCU values between the two germplasms, and there were only fluctuations in the single base level in a few codons. The number of termination codons (UAA, UAG, UGA) is the same as the RSCU value, and both prefer to use UAA (the number is 42, RSCU = 1.6155), with low preference for UGA (19, RSCU = 0.6537). Most amino acids show a tendency to use synonymous codons terminating in A/U, while the codons terminating in C/G are used less frequently, reflecting the obvious AT preference in this genome. The GC percentage is about 25–35%, belonging to the low GC type.

**Figure 2:**
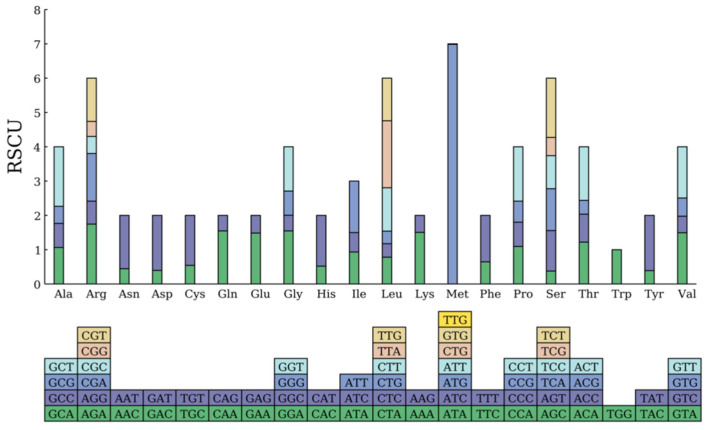
Histogram of wild *Corchorus olitorius* RSCU. The bar height above shows the overall RSCU value for all codons in that group, and the box represents all codons encoding each amino acid.

RSCU values for synonymous codons of different amino acid types were significantly differentiated (Figure 2). Most amino acids contained both preferred codons (RSCU > 1) and non-preferred codons (RSCU < 1), reflecting the selective utilization of specific synonymous codons by the species. The RSCU value of the start codon corresponding to methionine (MET) is 6.9827, which belongs to the strong preference codon. The RSCU of leucine (Leu) is also significantly different. Leu (Leu) UUA, alanine (ALA) GCU, arginine (ARG), serine (SER) UCU, aspartic acid (ASP) GGau, tyrosine (Tyr) UAU, and lysine (Lys) AAA show high preference for Rscu > 1. Because tryptophan (TRP) only corresponds to one coding codon of UGG, its RSCU value is 1, and there is no synonymous codon selection preference, which is in line except for codon degeneracy.

### 2.4. Analysis of Dispersed Repeat Sequences

**Figure 3:**
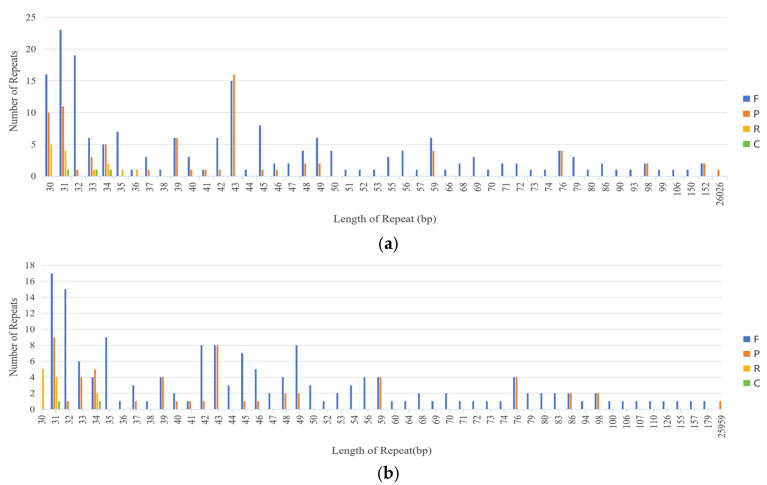
Statistical figures of QJYHM (**a**) and MLYHM (**b**) scattered repeats. The ordinate shows the frequency of distributed repeat sequences, while the abscissa shows their length. Forward repeats are indicated by F, palindromic repeats by P, reverse repeats by R, and complementary repeats by C.

The results of simple repeat sequence analysis of two wild species of jute showed that both sequences showed the rule of short sequence enrichment and long sequence sparseness. The core enrichment interval was 30–50 bp, and the quantity of sequences longer than 50 bp decreased significantly (Figure 3). One extremely long P-class sequence (26,026 bp and 25,959 bp, respectively) was detected in both sequences. The type composition showed a type bias of F/P class as the core, R/C class accounted for a very low proportion. Among them, QJYHM has 188 forward (F), 75 palindromes (P), 14 reverse (R), 3 complementary (C), and a total of 280 scattered repeats, and MLYHM has 175 forward (F), 64 palindromes (P), 11 reverse (R), 2 complementary (C), and a total of 252 scattered repeats (Table 4).

### 2.5. cpSSR Analysis

**Figure 4:**
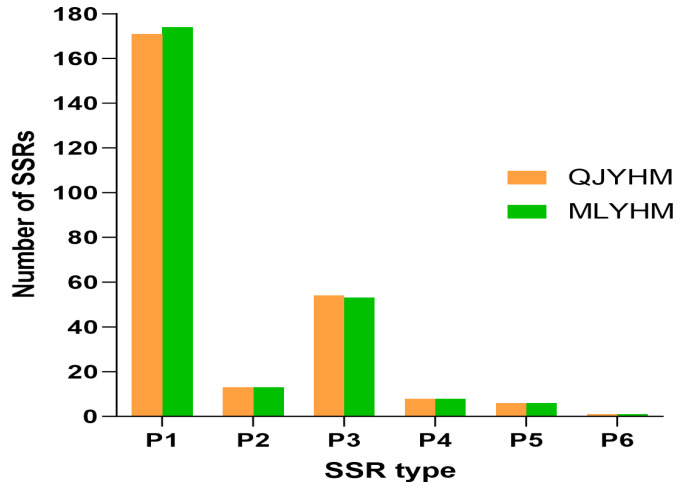
Distribution of wild *Corchorus olitorius* cpSSR marker types. CqSSR marker types are plotted on the abscissa, while the ordinate corresponds to the percentage of each marker type, and the data label is the number of CqSSR markers of this type.

The distribution of the SSR types of two wild species of jute with long fruit was analyzed. There were 253 and 255 SSR markers, respectively, and the species and number were very close (Figure 4). Both of them are dominant in single-nucleotide repeat (P1), accounting for 67.6% and 68.2%, respectively, indicating that single-nucleotide microsatellites are highly conserved in the jute genome. However, the number of single-nucleotide repeats (P1) in MLYHM was slightly higher than that in QJYHM, while the number of trinucleotide repeats (P3) was lower than that in QJYHM, which may reflect the structural variation in its genome in specific repeat regions. The percentage of dinucleotide repeat (P2) was relatively low in both of them, and the number was the same, suggesting that these markers were stable among varieties. Tetranucleotide (P4) and above repeat types were less in the two samples.

**Figure 5:**
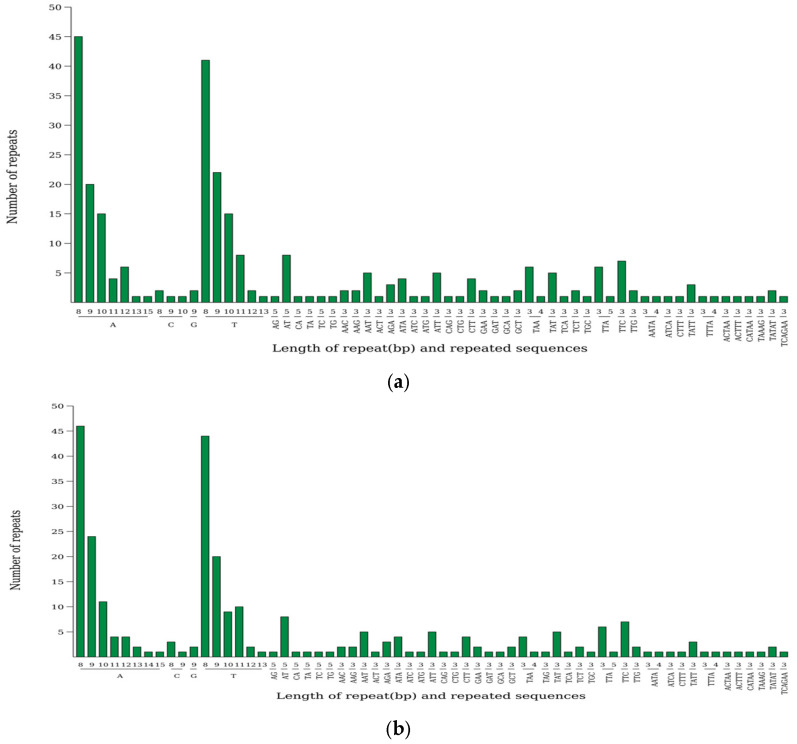
Quantitative statistics of SSR types in QJYHM (**a**) and MLYHM (**b**). The SSR repeat motif is plotted on the abscissa, while the ordinate represents the count of repeat units.

The composition patterns of SSR motifs in the mitochondrial genomes of the two tested accessions were highly consistent, with mononucleotide repeats showing a significant preference for A and T bases, exhibiting distinct AT enrichment; Among single-nucleotide SSRs, 8-bp short repeat fragments were the most abundant, with the number of loci gradually decreasing as the repeat length increased; among multi-nucleotide repeats, trinucleotides were the most abundant, while 4- to 6-nucleotide SSR loci were scarce. There was no significant differentiation in the distribution of loci across different repeat lengths among the genotypes, reflecting their close phylogenetic relationship and the high conservation of genomic SSR sequences (Figure 5).

### 2.6. KaKs Analysis

Based on the analysis of the Ka/Ks ratio, this study found that the whole chloroplast gene of jute was strongly purified (Ka/Ks < 1), indicating that its protein-coding sequence was highly conserved in the process of evolution. However, the *rpl23* gene showed a significant positive selection signal (Ka/Ks = 2.70335), indicating that the adaptive evolution of jute species seems to be significantly influenced by the ribosomal protein. In addition, unknown genes such as *ycf1* and *ycf2* also showed high Ka/KS (>1.6), which was worthy of further functional study. The great degree of similarity between MLYHM and QJYHM at the chloroplast genome level was further supported by the fact that there was no discernible difference in the Ka/Ks value of the chloroplast gene (Table 5). The reference sequences in Table 5 are chloroplast genome sequences of closely related species and cultivated varieties of the genus *Corchorus* from the NCBI database. Specifically, MW446503 and MZ522720 are from cultivated ramie (*Corchorus olitorius*), while MK251464 is from round-fruited ramie (*Corchorus capsularis*). These sequences were used to identify genes associated with adaptive evolution in wild ramie.

**Figure 6:**
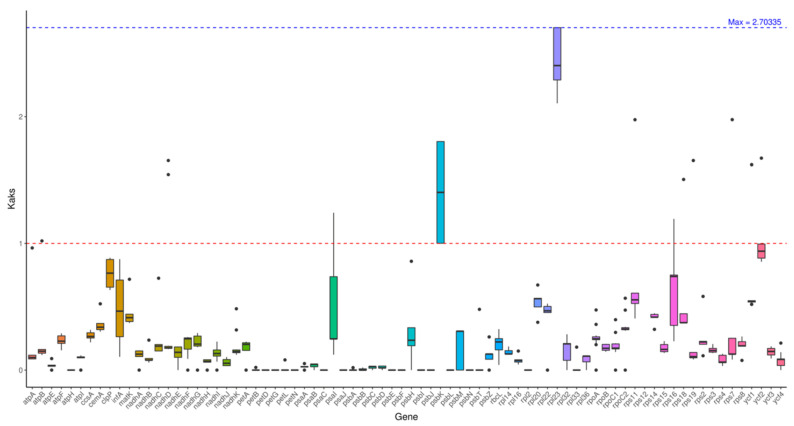
KaKs box diagram between species. The horizontal axis denotes gene names, while the vertical axis denotes Ka/Ks ratios. The red dashed line indicates that the Ka/Ks value is 1. For each boxplot, the upper and lower extremities of the whiskers correspond to the maximum and minimum observations. The box boundaries represent the first and third quartiles, with the median depicted as a bold line within the box. Outliers, defined as values exceeding 1.5 times the interquartile range, are plotted as individual dots.

Figure 6 reveals the characteristics of evolutionary selective pressure on jute chloroplast-encoded genes: Using the Asian QJYHM and African MLYHM materials as study subjects, the distributions of non-synonymous and synonymous substitution rates show that for the vast majority of chloroplast genes, these rates are far less than 1. In particular, core photosynthetic system genes, ATP synthase genes, and ribosomal protein genes generally fall within the range of 0–0.5, indicating that these genes are subject to strong purifying selection (negative selection), with highly conserved sequences and strictly constrained functions; only a few genes were significantly greater than 1, exhibiting clear signals of positive selection.

### 2.7. Joint Analysis of InDel and SNP

The entire chloroplast genome of the jute varieties QJYHM and MLYHM was examined for genetic variation. A total of 161 variation loci were found, including 97 SNPs and 64 InDels (Table 6). The functional gene area is comparatively conserved, and the variation is mostly found in the non-coding region (>85%) (Table 6). The transition/transversion ratio (Ts/Tv ≈ 1.77) obtained from SNP analysis conformed to the canonical pattern observed in plant chloroplast genomes. Some genes associated with photosynthesis and transcriptional regulation showed an enrichment of mutations; among them, the *ycf1* gene contained 5 SNPs and 1 InDel; In addition, the *ndhD*, *ndhF*, *ndhG*, *ndhI*, *ndhK*, *rpoA*, *rpoC1*, and *rpoC2* genes also carry multiple SNPs. The overall difference in the chloroplast genome between the two varieties was small, which indicated that they had high cytoplasmic compatibility as hybrid parents. The chloroplast genomes of these two species show little overall variation, and the two wild-type samples exhibit extremely low genetic variation (only 161 variant sites), indicating a high degree of intraspecific conservation.

### 2.8. IR Boundary Change Analysis

**Figure 7:**
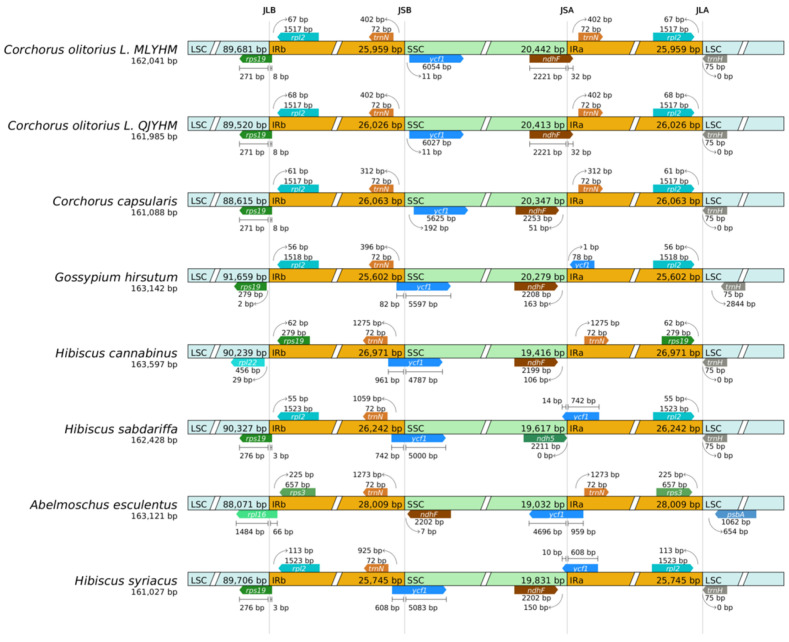
IR boundary change analysis diagram. Each region’s connection point is represented by a thin line, and the map displays the genes that are close to the connection point.

Nevertheless, there were notable species-specific differences in the length of each section (Figure 7). The composition of chloroplast genome regions of the two wild *Corchorus olitorius* species was highly conserved, and the difference was mainly due to the LSC region (the length difference was 161 bp). It is worth noting that *Abelmoschus esculentus* has the longest IR region (28,009 bp) and the shortest SSC region (19,032 bp), while *Gossypium hirsutum* has the longest LSC region (91,659 bp), forming a unique structural feature.

An examination of the IR boundary characteristics revealed that the *rps19* gene is positioned proximate to the large single-copy (LSC)/IRb boundary (JLB) in *Corchorus olitorius*. In contrast, this gene is entirely localized within the LSC region in *Gossypium hirsutum* and completely encompassed by the IRb region in Hibiscus cannabinus. Furthermore, in *Abelmoschus esculentus*, the LSC/IRb boundary (JLB) is characterized by the presence of the *rpl16* gene rather than the *rps19* gene. The *ndhF* genes of two wild *Corchorus olitorius* specimens span the IRa/SSC boundary (JSA), whereas no *ndhF* gene was detected in *Hibiscus sabdariffa*. In the remaining five species examined, the *ndhF* gene is located within the SSC region proximal to the IRa/SSC boundary (JSA). Typically, the *ycf1* gene extends across the IRb/SSC or IRa/SSC boundary. However, in *Corchorus*, it is situated entirely within the SSC region. The difference in the *ycf1* gene between the two wild *Corchorus olitorius* is 27 bp, and it is 402–429 bp from *Corchorus capsularis*, which belongs to the same genus. In addition, the size of the *ycf1* gene varies significantly among different genera within the Malvaceae family, ranging from 618 to 6054 base pairs, which is consistent with known characteristics of the Malvaceae family. The *trnN* genes of the eight Malvaceae species were all confined to the IR region, located precisely 72 bp from the junction, reflecting the high degree of structural stability characteristic of the Malvaceae chloroplast genome.

### 2.9. Homology and Collinearity Analysis of Chloroplast Sequences

**Figure 8:**
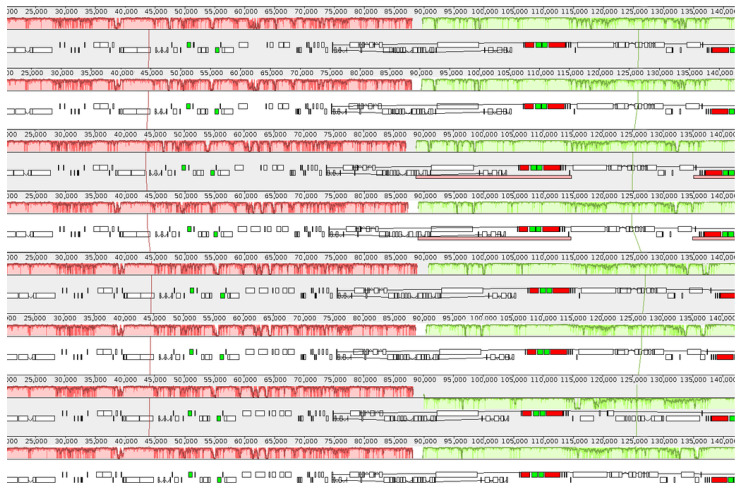
Homology analysis of chloroplast sequence. Gene locations are depicted by short boxes: white signifies CDS, green signifies tRNA, and red signifies rRNA. Locally Collinear Blocks (LCBs) are shown in color, indicating regions of homology aligned with another genome. Alignment blocks positioned above the central axis exhibit a forward orientation with respect to the reference genome, while those located below the axis display a reverse complement orientation. Intervening regions lack significant homology across the compared genomes. For each block, Mauve displays a similarity plot, with the vertical height reflecting the mean sequence similarity within that region. Links connecting the colored rectangles denote collinearity between genomic segments.

Mauve software (v2.3.1) was employed to assess chloroplast genome homology across multiple Malvaceae genera, including *Corchorus*, *Gossypium*, *Hibiscus*, and *Abelmoschus*. The analysis revealed high overall genomic conservation among the examined species (Figure 8). Local collinear regions (LCBs) distributed continuously in large areas cover almost all genome regions, and there are only a few scattered regions lacking homology. LCB blocks across different species show a high degree of consistency in terms of position and length; no obvious rearrangement events were observed in the gene sequences (coding regions, tRNA, rRNA), and the vast majority of LCB blocks are located above the centromere. Further analysis of the sequence similarity map in the LCB block shows that the similarity of each homologous region is highly evenly distributed, suggesting that the homologous sequence similarity of chloroplast genomes of different species is stable and high (Figure 8). At the intraspecific level, the chloroplast genome sequences and structures of different samples of *Corchorus olitorius* were almost identical, reflecting strong intraspecific conservation. Although the chloroplast genome among genera maintains the core collinearity and gene arrangement pattern, there are slight differences in the details of LCB in local regions, which is consistent with the general rule of chloroplast genome evolution conservation in Malvaceae groups. The extensively conserved syntenic regions within the chloroplast genome of wild *Corchorus olitorius* serve as important molecular markers for species identification and phylogenetic analysis, while the divergent regions represent potential targets for discovering novel genetic variation and enhancing parental diversity in breeding programs.

### 2.10. Phylogenetic Analysis

**Figure 9:**
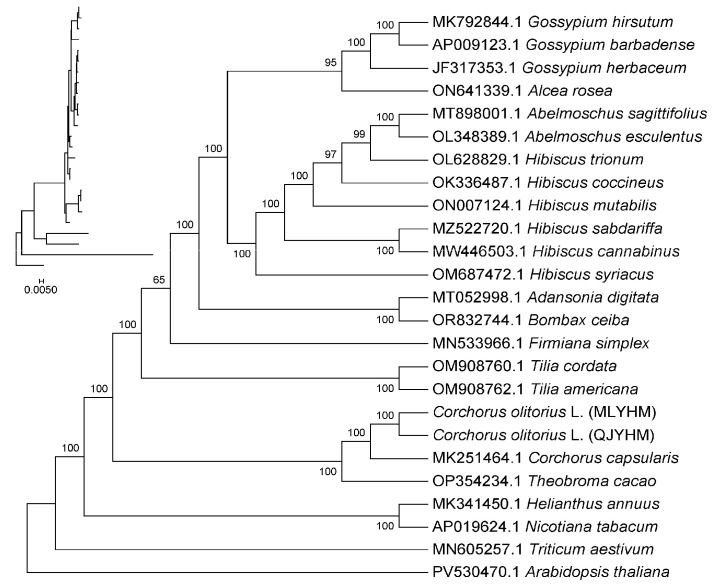
Phylogenetic tree of wild *Corchorus olitorius* and related Malvaceae species. (1) Sequence labels correspond to the Latin binomial of each species. (2) Branch length reflects genetic divergence; shorter branches indicate closer evolutionary relationships and smaller sequence differences. (3) The distance scale provides a unit of measurement for genetic divergence, analogous to a scale bar. (4) Bootstrap values ranging from 0 to 100 are presented at the nodes, representing the statistical confidence associated with each branch.

In order to determine the phylogenetic relationship of jute, the phylogenetic tree constructed by the chloroplast genome sequence clearly revealed the phylogenetic relationship of wild *Corchorus olitorius* in Malvaceae. The analysis shows that the self-development values of all key nodes are high, indicating that the topology has high statistical reliability (Figure 9). The two wild *Corchorus olitorius* species were closely clustered into one branch with 100% self-development value, and the branch length was very short, which confirmed that the genetic background of the two species was highly consistent. This species forms a sister group with *Corchorus capsularis* with extremely high support (100%), which is consistent with the known phylogenetic relationships within the genus *Corchorus*.

The genus Ramie, together with the genera *Hibiscus* and *Gossypium*, forms the core clade of the Malvaceae family, while species of the genus *Tilia* constitute a distinct peripheral clade, which is consistent with taxonomic conclusions.

## 3. Discussion

The chloroplast genome is highly conserved in terms of its structural organization and gene composition, making it a powerful tool for species identification, germplasm characterization, and phylogenetic inference [26]. However, in this study, we still identified minor differences during the assembly and annotation of the whole chloroplast genomes of two wild jute (*Corchorus olitorius*) accessions (QJYHM and MLYHM) from Asia and Africa. The chloroplast genome sizes of the two wild jute species analyzed in this study were 161,985 bp (QJYHM) and 162,041 bp (MLYHM), respectively, which differ from the chloroplast genome size (161,766 bp) previously reported for cultivated jute varieties [25].

Major changes at the level of chloroplast genome structure are manifested by a shortened LSC region, the folding of the IR boundary into the SSC, the *ycf15* pseudogene, and a reduction in simple repeat sequences. These results indicate that the chloroplast genome of jute has undergone detectable drift during the process of domestication. Furthermore, slight differences exist between the chloroplast genomes of cultivated jute in Asia and wild jute native to Africa. In MLYHM, the LSC and SSC are longer, while the IR is shorter, representing a typical IR contraction/expansion phenomenon in chloroplast genome evolution [27]. This provides partial evolutionary evidence at the organelle level for elucidating the geographical formation of the jute lineage and the rate of intraspecific differentiation [28].

Analysis of codon usage preferences revealed that both wild jute lines exhibited a strong preference for synonymous codons ending in A/U. This is a common feature of angiosperm chloroplast genomes and has been widely observed in hibiscus (*Hibiscus sabdariffa*), okra (*Abelmoschus esculentus*), and other Malvaceae plants [29]. This pattern is thought to be related to translational efficiency and environmental adaptation during plant evolution [30]. The strong preference for the AUG codon encoding methionine further supports the conservation of the translation initiation mechanism in higher plant chloroplasts [31].

Repetitive sequences are key drivers of genomic structural evolution [32]. Analysis of repetitive sequences in two wild *Corchorus olitorius* lines revealed that QJYHM contained 280 scattered repetitive sequences (188 forward repeats, 75 palindromic repeats, 14 reverse repeats, and 3 complementary repeats), while MLYHM contained 252 scattered repetitive sequences (175 forward repeats, 64 palindromic repeats, 11 reverse repeats, and 2 complementary repeats). In both materials, scattered repeats were dominated by forward (F) and palindromic (P) repeats, while reverse (R) and complementary (C) repeats accounted for an extremely low proportion; furthermore, repeat lengths were concentrated in the 30–50 bp** range. This pattern reflects a conserved structural strategy in chloroplast genomes: moderate repetitive elements help maintain genomic stability, while excessively long repetitive sequences are restricted to prevent structural rearrangements [33]. Similar results have been reported in the chloroplast genomes of kenaf (*Hibiscus cannabinus*) species, indicating that short repeat sequences and single-nucleotide SSRs are highly conserved in Malvaceae chloroplast genomes [34]. Although the low variation between the two varieties in this study limits the application of large-scale population genetic analysis or evolutionary inference, these cpSSR loci can still support basic germplasm identification.

Analysis of Ka/Ks selection pressures indicates that, in the chloroplast-encoded genes of the two wild *Corchorus olitorius* accessions studied here, the Ka/Ks ratios for the vast majority of genes are well below 1, suggesting that these genes have been subject to strong purifying selection. This is fully consistent with the highly conserved evolutionary pattern observed in essential genes responsible for core functions such as photosynthesis and self-replication in plant chloroplasts [35]. In this study, multiple genes, including *rpl23*, *ycf1*, and *ycf2*, exhibited significant signals of positive selection. Among them, the Ka/Ks ratio of the ribosomal large subunit protein gene *rpl23* reached as high as 2.70335, making it the site with the strongest positive selection; the Ka/Ks ratios for the conserved chloroplast open reading frame genes *ycf1* and *ycf2* were 1.62258 and 1.67323, respectively, both exhibiting distinct adaptive evolutionary characteristics. These genes are primarily involved in chloroplast ribosome assembly, gene translation regulation, and chloroplast development, and serve as key functional genes in plant responses to environmental stress [36].

A total of 161 variant loci (97 SNPs and 64 InDels) were identified between these two wild-type accessions, with *ycf1* emerging as a mutation hotspot. The *ycf1* gene has been confirmed as a highly variable region in the chloroplast genomes of many plant species and can serve as a core DNA marker for species identification [37].

The IR region exhibits only minor expansion and contraction; the IR lengths of QJYHM and MLYHM are 26,026 bp and 25,959 bp, respectively. The LSC/IRb (JLB) boundary is adjacent to the *rps19* gene, while the IRa/SSC (JSA) boundary is spanned by the *ndhF* gene. The *ycf1* gene is entirely located within the SSC region, and the *trnN* gene is stably located within the IR region, 72 bp from the boundary. These boundary characteristics fully align with the conserved evolutionary pattern of Malvaceae chloroplast genomes [38]. Comparison of IR boundaries and collinearity analysis indicate that the chloroplast genome structure of wild jute is highly consistent with that of other Malvaceae species, with no obvious gene rearrangement events detected [39].

## 4. Materials and Methods

### 4.1. Plant Materials

**Figure 10:**
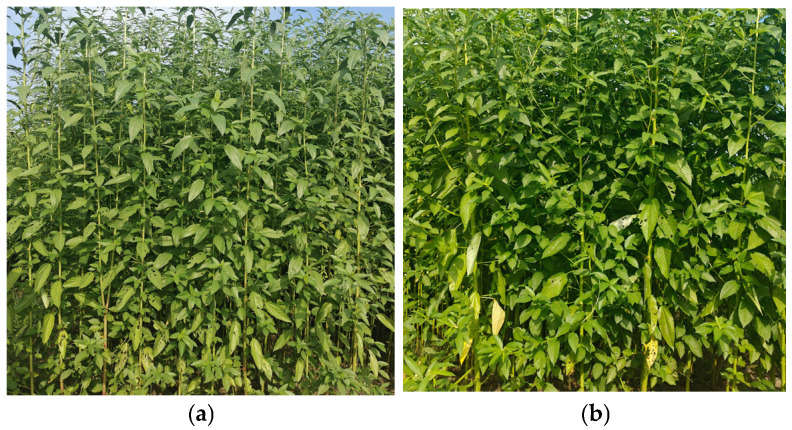
Field photographs of two wild *Corchorus olitorius* accessions used in the present study; (**a**) QJYHM, (**b**) MLYHM.

Fresh foliage from wild *Corchorus olitorius* varieties, namely Asian Qiaojianyehuangma (QJYHM) and African Maliyehuangma (MLYHM), served as the plant material for this investigation (Figure 10). Two accessions of wild *Corchorus olitorius* germplasm resources used in the research results came from the National Medium-Term Gene bank for Bast Fiber Crops. QJYHM was collected in Long’an County, Guangxi Province, China, numbered 00003121. MLYHM was collected from Mali, Africa, numbered 00000468. They were planted in the base at 30°4′24″ N, 120°13′19″ E, with an altitude of about 23.4 m. The experimental location is characterized by a subtropical climate, where the mean annual air temperature is 16.8 °C, the mean annual surface temperature is 17.7 °C, the annual precipitation totals 1440.5 mm, and the annual sunshine duration reaches 1804.6 h. The soil, classified as sandy loam, originated from the Qiantang River reclamation project and exhibited a pH value of 7.69.

The young leaves of healthy plants were washed to remove surface impurities and dried with clean filter paper to ensure no pollutant residues. A 2 g aliquot of each sample was precisely weighed, sealed in a pre-cooled microcentrifuge tube, and immediately cryopreserved by immersion in liquid nitrogen for 10 min. The frozen samples were subsequently maintained at −80 °C freezer for downstream applications.

### 4.2. Extraction and Sequencing of DNA

Genomic DNA was extracted using the Universal Plant DNA Kit (Jisi Huiyuan, Nanjing, Jiangsu, China, Cat. No. D312). Subsequent to quality assessment, the DNA was subjected to random fragmentation using a Covaris ultrasonic processor (Xiaomei Ultrasonic Instrument (Kunshan) Co., Ltd., Kunshan, Jiangsu, China). The fragmented DNA was then purified, end-repaired, A-tailed, and ligated with an adaptor. Target fragments were isolated using agarose gel electrophoresis before being amplified using PCR for library creation. The constructed library underwent a quality evaluation, and upon successful validation, paired-end sequencing (PE150) was performed using the Illumina Novaseq X Plus platform. Fastp (v0.23.4) was employed to process and filter the raw sequencing data for quality assurance.

### 4.3. Statistical Analysis

In this study, the obtained data were collated and analyzed by Excel 2019 software, and the histogram was drawn by Graphpad Prism 10.1.2 software. Chloroplot software (https://irscope.shinyapps.io/Chloroplot/ accessed on 25 July 2025) was used to draw the chloroplast genome map, and vmatch (v2.3.0) (http://www.vmatch.de/ accessed on 25 July 2025) software was used to identify the repeat sequences. The cpSSR analysis was performed using MISA (v1.0) (MIcroSAtellite identification tool, http://pgrc.ipk-gatersleben.de/misa/misa.html accessed on 30 July 2025) software. Gene sequence alignment was performed using mafft (v7.310) (https://mafft.cbrc.jp/alignment/software/ accessed on 5 August 2025) software. The Ka/Ks value of the gene was calculated using KaKs Calculator (v2.0) (https://sourceforge.net/projects/kakscalculator2/ accessed on 5 August 2025) software. The homologous gene sequences of different species were compared globally by mafft (v7.310) software (-auto mode). The CPJSdraw software was used to visualize the boundary information. The sequences between species were aligned by MAFFT software (v7.427, -auto mode). The aligned data were used to construct the maximum likelihood phylogenetic tree with RAxML (v8.2.10) (GTRGAMMA model in https://cme.h-its.org/exelixis/software.html accessed on 10 August 2025) software. Genomic alignment was performed using the default parameters of Mauve (v2.3.1) (https://darlinglab.org/mauve/mauve.html accessed on 10 August 2025) software.

### 4.4. Genome Assembly and Annotation

The sequencing data were aligned to the self-built chloroplast genome database using the Bowtie2 (v2.2.4) very-sensitive-local model to extract the chloroplast DNA (cpDNA) sequence. GetOrganelle (v1.7.7.1) was used for de novo assembly, and the kmer parameters were set to 55, 87, 121, independent of the reference genome. The assembly process includes obtaining seed sequences, iterative extension, SSPACE connection contig, GapFiller filling the gap, genome correction and coordinate rearrangement to obtain a complete circular chloroplast genome sequence.

Gene annotation was performed using a dual-annotation cross-validation strategy: Method 1: Prodigal (v2.6.3) was used to annotate the coding sequence (CDS) [40], hmmer (v3.1b2) was used to predict rRNA [41], and aragorn (v1.2.38) was used to predict tRNA [42]. Method 2: Based on the gene sequences of related species in NCBI, the homologous annotation results were obtained by blast (v2.6) alignment assembly sequence [43]. Manually verify the annotation results obtained by the two methods, eliminate redundant and incorrect annotations, and clarify the boundaries of multiple exons, and finally form a reliable annotation set. The chloroplast genome map was drawn by OGDRAW [44].

### 4.5. Procedures of RSCU Analysis

The Perl script written by Jisi Huiyuan Company is used for analysis. Initially, a single representative CDS was selected from each set of multi-copy CDS (only one copy was retained), and the RSCU values were subsequently computed using the established formula. For each codon, the RSCU value was derived by dividing its actual occurrence by the theoretically expected occurrence. The latter is given by the inverse of the total number of synonymous codons encoding the same amino acid.

### 4.6. Analysis of Dispersed Repetitive Sequences

The software vmatch (2.3.0) and Perl scripts were used to identify the repetitive sequences [45]. The parameters are set as follows: the minimum length is 30 bp, and the hamming distance is 3. Identification covers four types, including forward, palindromic, reverse, and complement. The number and type distribution of repetitive sequences with different lengths were further counted.

### 4.7. KaKs Analytical Methods

The MAFFT v7.427 [46] software was used to perform multiple-sequence alignment on the gene sequence, and then KaKs_Calculator v2.0 [47] was used to calculate the ratio of non-synonymous mutation rate (Ka) to synonymous mutation rate (Ks) (Ka/Ks). The parameter was set as -m MLWL-c 11 (11-Bacterial and Plant Plastid Code) to assess the selection pressure on genes.

### 4.8. Analysis of and Delimitation

Aiming at the analysis of chloroplast genome boundary structure, four boundary regions, LSC-IRb, IRb-SSC, SSC-IRa and IRa-LSC, were visualized by using the CPJSdraw tool in the Jisi Huiyuan biological cloud platform combined with Perl + SVG drawing script.

### 4.9. Phylogenetic Analysis Methods

In this study, 23 different genera of plants were selected, and Arabidopsis thaliana was used as an outgroup species to download the corresponding sequences from the NCBI database. PhyloSuite v1.2.3 was used to extract common gene sets from each sequence. Multiple sequence alignment was performed by MAFFT v7.427 (parameter set to-auto mode), trimAl (v1.4.rev15) [48] was used to trim the unreliable region by default conservative pruning parameters, and then the CDS sequences of each species were connected in series as a whole sequence. In the model selection stage, based on the Bayesian Information Criterion (BIC), jModelTest v2.1.10 was used to screen the optimal nucleotide substitution model. Finally, using RAxML v8.2.10 [49] and GTRGAMMA as the surrogate model, the maximum likelihood method with 1000 rapid Bootstrap replicates was used to construct the phylogenetic tree.

## 5. Conclusions

In this study, the complete chloroplast genomes of two wild jute (*Corchorus olitorius*) accessions from Asia and Africa (QJYHM and MLYHM) were sequenced. Both chloroplast genomes exhibited a typical conserved tetrad structure, with identical gene numbers (129) and GC content (36.76%), indicating a high degree of intraspecific conservation.

Codon usage preferences were dominated by A/U-terminated codons, and repetitive sequences consisted primarily of cis- and palindromic repeats, with single-nucleotide cpSSRs being the predominant type. Most chloroplast genes were subject to strong purifying selection, while a few genes (such as *rpl23*, *ycf1*, and *ycf2*) showed preliminary signals of positive selection. A total of 161 variant sites were detected, with *ycf1* being a mutation hotspot.

IR boundary and collinearity analyses confirmed that the chloroplast genomes of wild jute within the Malvaceae family exhibit high structural conservation. Phylogenetic analysis supports a close relationship between wild *Corchorus olitorius* and *Corchorus capsularis*, consistent with the known taxonomic status of the genus *Corchorus*.

This study provides foundational chloroplast genomic data for wild *Corchorus olitorius*, reveals its conserved genomic characteristics, and clarifies its phylogenetic position. Due to the small sample size, the results are limited and cannot support broad evolutionary or breeding conclusions; however, they lay the groundwork for future studies with expanded sampling of germplasm resources.

## Figures and Tables

**Table 1:** Chloroplast genome characteristics of wild *Corchorus olitorius*.

Features	QJYHM	MLYHM
Total genome size (bp)	161,985	162,041
LSC region length (bp)	89,520	89,681
SSC region length (bp)	20,413	20,442
IR region length (bp)	26,026	25,959
Overall GC content (%)	36.76	36.76
GC in LSC (%)	34.82	34.79
GC in SSC (%)	30.99	31.00
GC in IR (%)	42.35	42.43

**Table 2:** Summary of chloroplast gene functional categories in wild *Corchorus olitorius*.

Category	Gene Group	Gene Name	Number of Genes
Photosynthesis	Subunits of photosystem I	*psaA*, *psaB*, *psaC*, *psaI*, *psaJ*	5
	Subunits of photosystem II	*psbA*, *psbB*, *psbC*, *psbD*, *psbE*, *psbF*, *psbH*, *psbI*, *psbJ*, *psbK*, *psbL*, *psbM*, *psbN*, *psbT*, *psbZ*	15
	Subunits of NADH dehydrogenase	ndhA*, ndhB*(2), *ndhC*, *ndhD*, *ndhE*, *ndhF*, *ndhG*, *ndhH*, *ndhI*, *ndhJ*, *ndhK*	12
	Subunits of the cytochrome b/f complex	*petA*, petB*, petD*, *petG*, *petL*, *petN*	6
	Subunits of ATP synthase	*atpA*, *atpB*, *atpE*, atpF*, *atpH*, *atpI*	6
	Large subunit of rubisco	*rbcL*	1
	Subunits of photochlorophyllide reductase	*-*	0
Self-replication	Proteins of large ribosomal subunit	*rpl14*, rpl16*, rpl2*(2), *rpl20*, *rpl22*, *rpl23*(2), *rpl32*, *rpl33*, *rpl36*	11
	Proteins of small ribosomal subunit	*rps11*, rps12**(2), *rps14*, *rps15*, rps16*, *rps18*, *rps19*, *rps2*, *rps3*, *rps4*, *rps7*(2), *rps8*	12
	Subunits of RNA polymerase	*rpoA*, *rpoB*, rpoC1*, *rpoC2*	4
	Ribosomal RNAs	*rrn16*(2), *rrn23*(2), *rrn4.5*(2), *rrn5*(2)	8
	Transfer RNAs	trnA-UGC*(2), *trnC-GCA*, *trnD-GUC*, *trnE-UUC*, *trnF-GAA*(2), trnG-GCC*, *trnH-GUG*, *trnI-CAU*(2), trnI-GAU*(2), trnK-UUU*, *trnL-CAA*(2), trnL-UAA*, *trnL-UAG*, *trnM-CAU*, *trnN-GUU*(2), *trnP-UGG*, *trnQ-UUG*, *trnR-ACG*(2), *trnR-UCU*, *trnS-GCU*, *trnS-GGA*, *trnS-UGA*, *trnT-GGU*, *trnT-UGU*, *trnV-GAC*(2), trnV-UAC*, *trnW-CCA*, *trnY-GUA*, *trnfM-CAU*	40
Other genes	Maturase	*matK*	1
	Protease	clpP**	1
	Envelope membrane protein	*cemA*	1
	Acetyl-CoA carboxylase	*-*	0
	c-type cytochrome synthesis gene	*ccsA*	1
	Translation initiation factor	*infA*	1
	other	*-*	0
Genes of unknown function	Conserved hypothetical chloroplast ORF	*ycf1*, *ycf2*(2), ycf3**, *ycf4*	5
Total			129

Genes with one intron are indicated by an asterisk (*); genes with two introns are indicated by double asterisks (**); genes with multiple copies are indicated by parentheses (2), which also display the copy number.

**Table 3:** RSCU values characteristics of wild *Corchorus olitorius*.

Symbol	Codon	The Number of QJYHM Codons	RSCU of QJYHM	The Number of MLYHM Codons	RSCU of MLYHM
Ter	UAA	42	1.6155	42	1.6155
Ter	UAG	19	0.7308	19	0.7308
Ter	UGA	17	0.6537	17	0.6537
Ala	GCA	339	1.0652	339	1.0652
Ala	GCC	223	0.7008	224	0.704
Ala	GCG	159	0.4996	158	0.4964
Ala	GCU	552	1.7344	552	1.7344
Cys	UGC	67	0.5426	67	0.5426
Cys	UGU	180	1.4574	180	1.4574
Asp	GAC	182	0.396	183	0.397
Asp	GAU	737	1.604	739	1.603
Glu	GAA	865	1.4824	864	1.482
Glu	GAG	302	0.5176	302	0.518
Phe	UUC	424	0.6474	424	0.6478
Phe	UUU	886	1.3526	885	1.3522
Gly	GGA	607	1.5476	606	1.546
Gly	GGC	179	0.4564	178	0.454
Gly	GGG	276	0.7036	277	0.7068
Gly	GGU	507	1.2924	507	1.2932
His	CAC	137	0.521	136	0.518
His	CAU	389	1.479	389	1.482
Ile	AUA	610	0.9357	610	0.9351
Ile	AUC	367	0.5628	367	0.5625
Ile	AUU	979	1.5015	980	1.5024
Lys	AAA	900	1.5062	902	1.5084
Lys	AAG	295	0.4938	294	0.4916
Leu	CUA	306	0.78	306	0.7794
Leu	CUC	155	0.3954	155	0.3948
Leu	CUG	143	0.3648	143	0.3642
Leu	CUU	496	1.2648	497	1.266
Leu	UUA	765	1.9506	766	1.9506
Leu	UUG	488	1.2444	489	1.2456
Met	AUA	0	0	0	0
Met	AUC	0	0	0	0
Met	AUG	515	6.9867	515	6.9867
Met	AUU	1	0.0133	1	0.0133
Met	CUG	0	0	0	0
Met	GUG	0	0	0	0
Met	UUG	0	0	0	0
Asn	AAC	243	0.4442	243	0.4438
Asn	AAU	851	1.5558	852	1.5562
Pro	CCA	255	1.0944	257	1.1032
Pro	CCC	165	0.708	165	0.708
Pro	CCG	143	0.6136	141	0.6052
Pro	CCU	369	1.5836	369	1.5836
Gln	CAA	622	1.5454	622	1.5454
Gln	CAG	183	0.4546	183	0.4546
Arg	AGA	393	1.7454	394	1.7484
Arg	AGG	151	0.6708	151	0.6702
Arg	CGA	313	1.3902	312	1.3848
Arg	CGC	111	0.4932	112	0.4968
Arg	CGG	99	0.4398	99	0.4392
Arg	CGU	284	1.2612	284	1.2606
Ser	AGC	103	0.3768	102	0.3738
Ser	AGU	322	1.179	322	1.1802
Ser	UCA	334	1.2228	334	1.224
Ser	UCC	263	0.963	263	0.9642
Ser	UCG	145	0.531	145	0.5316
Ser	UCU	472	1.728	471	1.7262
Thr	ACA	358	1.2196	358	1.2156
Thr	ACC	240	0.8176	241	0.8184
Thr	ACG	118	0.402	118	0.4008
Thr	ACU	458	1.5604	461	1.5652
Val	GUA	454	1.4936	455	1.4968
Val	GUC	147	0.4836	146	0.4804
Val	GUG	161	0.5296	161	0.5296
Val	GUU	454	1.4936	454	1.4936
Trp	UGG	397	1	397	1
Tyr	UAC	161	0.3918	161	0.3912
Tyr	UAU	661	1.6082	662	1.6088

Symbol: Three-letter abbreviation of amino acid.

**Table 4:** Statistics of scattered repeats in wild *Corchorus olitorius*.

#ID	F	P	R	C	Total
QJYHM	188	75	14	3	280
MLYHM	175	64	11	2	252

Forward, palindromic, reverse, and complementary repeats are indicated by F, P, R, and C, respectively.

**Table 5:** Genes identified as being under positive selection based on Ka/Ks ratios > 1.

Gene	Functional Categories	Maximum Ka/Ks Value	Reference Sequence (NCBI Accession)
*rpl23*	Large ribosomal subunit protein	2.70335	MW446503/MZ522720
*rps11*	Small ribosomal subunit protein	1.97667	MK251464
*ycf1*	Hypothetical chloroplast protein	1.62258	MK251464
*ycf2*	Hypothetical chloroplast protein	1.67323	MK251464
*rps7*	Small ribosomal subunit protein	1.97730	MK251464
*rps18*	Small ribosomal subunit protein	1.50505	MK251464
*rps19*	Small ribosomal subunit protein	1.65455	MK251464
*nadhD*	NADH dehydrogenase subunit D	1.65463	MK251464

Note: Gene refers to the identification number assigned to each of the two sequences. A Ka/Ks ratio greater than 1 indicates that the gene is subject to positive selection; the reference sequences are the chloroplast genomes of cultivated and closely related species of the genus Jute, as listed in the NCBI database.

**Table 6:** Summary of Key Variant Sites.

Variant Type	Total	In Genes	Intergenic	Functional Genes Impacted
SNP	97	21	76	*ycf1*(5), *ndh cluster*(6)
InDel	64	1	63	*ycf1*(1)
Total	161	22	139	Predominantly photosynthesis-related

## Data Availability

All sequences and annotation data were deposited in a public database at the National Center for Biotechnology Information (https://www.ncbi.nlm.nih.gov/) under accession numbers PX636893 and PX633638.
